# 2-{3-Cyano-4-[2-(4-diethylamino-2-hy­droxyphenyl)ethenyl]-5,5-dimethyl-2,5-dihydrofuran-2-ylidene}malononitrile acetone 0.25-solvate

**DOI:** 10.1107/S1600536812039736

**Published:** 2012-09-26

**Authors:** Graeme J. Gainsford, Mohamed Ashraf, Andrew J. Kay

**Affiliations:** a Industrial Research Limited, PO Box 31-310, Lower Hutt, New Zealand

## Abstract

In the title compound, C_22_H_22_N_4_O_2_·0.25C_3_H_6_O, the disordered acetone mol­ecule lies with partial occupancy about the 2 axis. The mol­ecule of the malononitrile derivative is essentially planar excluding the methyl groups, with the largest deviation from the mean plane through the non-H atoms being 0.1955 (13) Å. Two rotamers with different orientations of the benzene ring are observed in the ratio of 0.919 (2):0.081 (2), and as a result the OH group is disordered over two sets of sites. In the crystal, the mol­ecules form ribbons along (101) utilizing a strong O—H⋯N(cyano) hydrogen bond. Inter­leaving of the nearly planar ribbons is provided by the twofold disordered acetone molecule through C—H⋯O inter­actions.

## Related literature

For organic push–pull conjugated molecules in electro-optical applications, see: Dalton (2004 ▶); Ma *et al.* (2002 ▶); Marder *et al.* (1997 ▶); Li *et al.* (2007 ▶); Avetisyan *et al.* (2009 ▶); Gainsford *et al.* (2008 ▶). For related structures, see: Li *et al.* (2009 ▶); Wu *et al.* (2012 ▶). For the Cambridge Structural Database, see: Allen (2002 ▶).
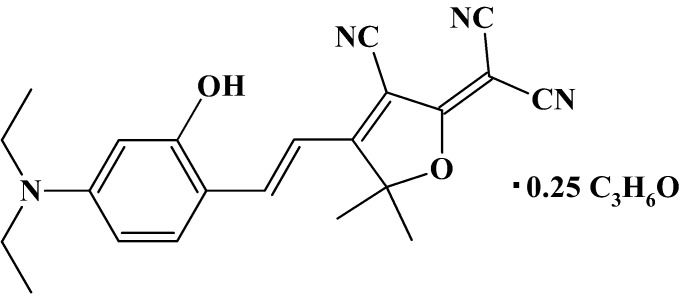


## Experimental

### 

#### Crystal data

4C_22_H_22_N_4_O_2_·C_3_H_6_O
*M* *_r_* = 1555.82
Monoclinic, 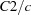

*a* = 18.6899 (6) Å
*b* = 14.4941 (4) Å
*c* = 16.7485 (5) Å
β = 95.266 (2)°
*V* = 4517.9 (2) Å^3^
*Z* = 2
Mo *K*α radiation
μ = 0.08 mm^−1^
*T* = 113 K
0.61 × 0.53 × 0.33 mm

#### Data collection

Bruker-Nonius APEXII CCD diffractometer
Absorption correction: multi-scan (*SADABS*; Blessing, 1995 ▶) *T*_min_ = 0.678, *T*_max_ = 0.746
51842 measured reflections
6086 independent reflections
4957 reflections with *I* > 2σ(*I*)
*R*_int_ = 0.033

#### Refinement

*R*[*F* ^2^ > 2σ(*F* ^2^)] = 0.047
*wR*(*F* ^2^) = 0.138
*S* = 1.04
6089 reflections
289 parameters
5 restraints
H atoms treated by a mixture of independent and constrained refinement
Δρ_max_ = 0.44 e Å^−3^
Δρ_min_ = −0.34 e Å^−3^

### 

Data collection: *APEX2* (Bruker, 2005 ▶); cell refinement: *SAINT* (Bruker, 2005 ▶); data reduction: *SAINT* and *SADABS* (Bruker, 2005 ▶); program(s) used to solve structure: *SHELXS97* (Sheldrick, 2008 ▶); program(s) used to refine structure: *SHELXL97* (Sheldrick, 2008 ▶); molecular graphics: *ORTEP-3* (Farrugia, 1997 ▶) and *Mercury* (Macrae *et al.*, 2008 ▶); software used to prepare material for publication: *SHELXL97* and *PLATON* (Spek, 2009 ▶).

## Supplementary Material

Crystal structure: contains datablock(s) global, I. DOI: 10.1107/S1600536812039736/yk2072sup1.cif

Structure factors: contains datablock(s) I. DOI: 10.1107/S1600536812039736/yk2072Isup2.hkl

Supplementary material file. DOI: 10.1107/S1600536812039736/yk2072Isup3.cml

Additional supplementary materials:  crystallographic information; 3D view; checkCIF report

## Figures and Tables

**Table 1:** Hydrogen-bond geometry (Å, °)

*D*—H⋯*A*	*D*—H	H⋯*A*	*D*⋯*A*	*D*—H⋯*A*
O2*A*—H2O*A*⋯N1^i^	0.88 (2)	1.96 (2)	2.8095 (16)	162 (2)
C17—H17⋯O3	0.95	2.37	3.213 (13)	148
C17—H17⋯O3^ii^	0.95	2.51	3.324 (13)	144

Symmetry codes: (i) 
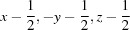
; (ii) 
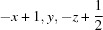
.

**Fig. 1.**
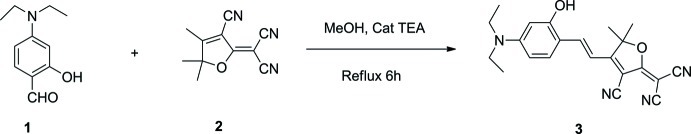
Chemical synthesis of the title compound 3.

**Fig. 2.**
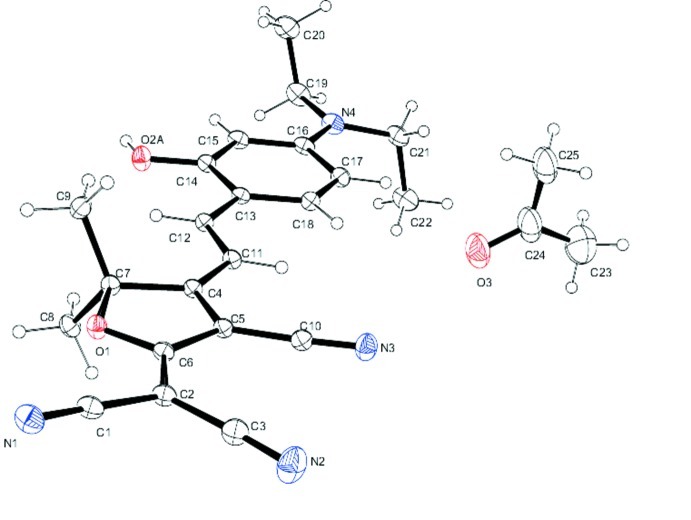
Structure of the asymmetric unit (Farrugia, 1997) showing the atom labelling scheme and displacement ellipsoids drawn at the 30% probability level. Only the major rotamer atom (O2A) is shown (see text).

**Fig. 3.**
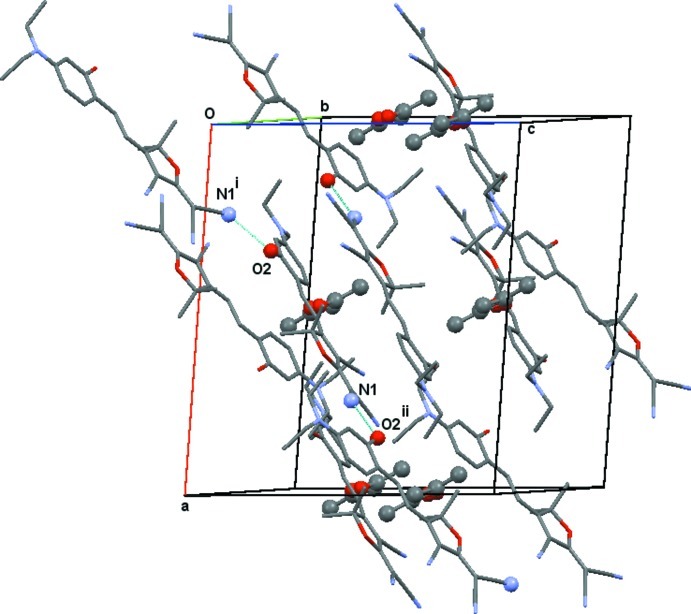
Packing diagram (Macrae et al., 2008) of the unit cell. H atoms excluded for clarity. Disordered acetone and H bonding atoms shown as balls. Close contacts indicated by dotted lines identify the key H bond (see text). Symmetry (i) -1/2 + x, -1/2 - y, -1/2 + z (ii) 1/2 + x, -1/2 - y, 1/2 + z.

## supplementary crystallographic information

### Comment

Organic donor–π-acceptor (D–π-A) molecules show much promise due to their potential application in areas such as photonics, optical power limiting and optical data storage (Dalton, 2004; Ma *et al.*, 2002). These molecules are typically push–pull conjugated systems that can be modified by altering either the donor, acceptor or conjugated interconnect moieties. Consequently, there are typically a number of options available to iteratively improve the overall molecular response and stability of such compounds, especially when applied to their use in second-order nonlinear optics. For example, a successful approach to optimizing their second-order nonlinear optical (NLO) response is based on tuning the ground-state polarization - and hence the degree of bond-length alternation - through modification of the end groups and the spacer (Marder *et al.*, 1997). Furthermore, thermal and photochemical stability can be improved through the use of ring-locked spacer units between the donor–acceptor moieties. However, in order to be successfully deployed in devices the chromophores need to be embedded into a polymer and their dipoles aligned in a non-centrosymmetric fashion using a process known as poling. This can be difficult to achieve as NLO chromophores embedded in polymer matrices have a tendency to aggregate due to their large dipole moments. As a result there is often a need to find expedient methods to minimize aggregation in NLO chromophores and these include the use of bulky fluorinated and non-fluorinated pendant groups as well as the use of hydrogen-bonding substituents to control the molecular interactions.

Studies of hydrogen bonds connecting organic and organic–inorganic compounds have long been a topic of intense research in crystal engineering because this allows not only for a rational approach to bottom-up construction but hydrogen bonds also effectively regulate the molecular architecture (Li *et al.*, 2007). With this in mind, and in line with our on-going work on the development of novel organic NLO compounds, we sought a straightforward route to a D–π-A molecule containing a hydroxyl substituent to allow us to study its impact on crystal packing, as well as providing a potentially reactive site for future modifications. Consequently, we prepared the title compound 3 using the method outlined in Fig. 1. This involved the condensation of 5-diethylaminosalicylaldehyde (1) with 2-(3-cyano-4,5,5-trimethyl-5*H*-furan-2-ylidene)-malononitrile 2 and provided the title compound in an 80% yield. Compound 2 was prepared by the general procedure reported in the literature (Avetisyan *et al.*, 2009).

Compound REFCODES are from the C.S.D. (Version 5.33, with May 2012 updates; Allen, 2002). The molecule is rotationally disordered about the C12—C13 bond in the ratio of 0.919 (2):0.081 (2) as determined by refining of the O2—H atoms over two positions with restraints (see experimental). Refinement of the remaining rotamer atoms was not practical at the ~8% level. The asymmetric unit contents of the major rotamer of the title compound (I) are shown in Fig. 2.

The 5-membered ring plane of atoms O1,C4–C7 (hereafter `CDFP', [3-cyano-5,5-dimethyl-2,5-dihydrofuran-2-ylidene]propanedinitrile) is planar with maximum out of plane deviation for O1 of 0.003 (1) Å. The dicyano group (N1,C1,C2,C3,N2,C6) is planar but twisted by 5.46 (6)° with respect to the `CDFP' group as been found in previous studies *e.g.* 5.69 (17)° in compound NOJKUT (Gainsford *et al.*, 2008). The entire `backbone' including the hydroxyl atom O2 and C2 but excluding the ethyl and methyl atoms (C8,C9,C19–C22) and the dicyano groups can be considered essentially planar with average mean plane deviation 0.019 (1) Å and maximum deviation 0.027 (1) Å for C11. This is in marked contrast to the related benzoyloxy structure KARXAE (Wu *et al.*, 2012) ((4-(2(2-Benzoyloxy)-4-(diethylamino)phenyl)vinyl)-3-cyano-5,5 -dimethylfuran-2(5*H*)-ylidene) malonitrile), where the CDFP and (diethylamino)phenyl groups make interplanar angles of ~10°. The difference probably relates to the different crystal packing arrangements, as noted below, but this twist also alleviates potential close contacts between the methylene group of the benzoyloxy group and the pendant nearest ethyl group. The pendant ethyl groups in KARXAE are also in the opposite configurations, with the nearest ethyl group pointing away from the benzoyloxy moiety. The acetone molecule is disordered around a 2 fold axis, and the final concentration was decided by a thermal parameter comparison with the other atoms and confirmed by a stable final refinement.

One other related structure, without the *ortho* oxygen substituent at C15, is NUGNUZ (Li *et al.*, 2009). Here the backbone is twisted along its length, with ~7° between CDFP and the polyene atoms, and a further ~ 8° between the latter and the (diethylamino)phenyl group. This twisting is probably driven by the intermolecular hydrogen bonding interactions which involve one of the polyene H atoms (to the terminal alcohol O) and a phenyl C—H···N(cyano) contact.

The crystal packing can be described as interleaved ribbons of molecules, approximately in the 1,0,1 direction, formed by the major almost in-plane hydrogen bonding (entry 1, Table 1 and Figure 3). The alternate ribbon molecule planes make a dihedral angle of ~15°. The interplanar interactions are provided by weak C—H···O(acetone) interactions (entries 2 and 3, Table 1). Given this weak interaction (and the estimated 0.25 concentration of the acetone), it is not surprising that rotational conformers are present. By contrast the packing in related structure KARXAE is a traditional herringbone pattern, with ~39° between the molecular planes; here the single weak C—H···N(cyano) and (methylene)C–H···π are sufficient for the crystal packing stability.

### Experimental

To a stirred solution of 5-diethylaminosalicyladehyde 1 (0.97 g, 5 mmol) in methanol was added compound 2 (0.99 g, 5 mmol) (Fig. 1). Two drops of triethylamine were then added and the mixture was then refluxed for 6 h, by which time its colour had changed to deep violet. The solid formed was filtered and purified by recrystallization from ethanol to give the titled compound 3 as a violet solid (1.49 g, 80% yield). X-ray quality crystals were grown by slow evaporation from acetone. m.p. 223.8°C. ^1^H NMR (500 MHz, DMSO): δ 10.80 (s, 1H), 8.21 (d, 1H, *J* 15 Hz), 7.69 (d, 1H, *J* 10 Hz), 6.94 (d, 1H, *J* 15 Hz), 6.45 (d, 1H, *J* 10 Hz), 6.17 (s, 1H), 3.45 (q, 4H), 1.70 (s, 6H), 1.16 (t, 6H). ^13^C NMR (75 MHz, CDCl_3_): δ 177.50, 175.32, 162.21, 154.08, 145.02, 114.03, 113.16, 112.79, 111.99, 107.02, 106.77, 97.18, 96.50, 48.80, 44.54, 25.93, 12.65. LCMS Found: MNa^+^ 397.1643; C_22_H_22_N_4_O_2_Na requires MNa^+^ 397.1640; Δ = 0.8 p.p.m.

### Refinement

The molecule is rotationally disordered about the C12—C13 bond in the ratio of 0.919 (2):0.081 (2) as determined by refining the O2—H atoms over two positions with identical thermal parameters (EADP). Four further restraints were applied to the minor rotamer atoms with C18—O2B and O2B—H2B fixed at 1.340 (5) and 0.84 (1) Å respectively, and two antibumping restraints tied to the equivalent major rotamer distances (using SADI). The final residual difference density is consistent with this ~8% presence for the remaining atoms in the rotated group; their refinement is impractical and would add nothing to the final conclusions.

Four reflections affected by the backstop and 16 others, which were clearly outlier data (mostly at low angle) with Δ(F^2^)/e.s.d. > 5.0, were omitted from the refinements (using OMIT). The methyl and other H atoms were refined with *U*_iso_ 1.5 and 1.2 times respectively that of the *U*_eq_ of their parent atom. The hydroxyl hydrogen on major rotamer O2A was located on a difference Fourier map and its position refined. The hydroxyl hydrogen bound to the (8%) O2B atom was located *via* an HFIX 147 tetrahedral position refinement and refined as noted above. All H atoms bound to carbon were constrained to their expected geometries (C—H 0.95, 0.98 and 0.99 Å).

### Crystal data

**Table:**

4C_22_H_22_N_4_O_2_·C_3_H_6_O	*F*(000) = 1648
*M**_r_* = 1555.82	*D*_x_ = 1.144 Mg m^−^^3^
Monoclinic, *C*2/*c*	Mo *K*α radiation, λ = 0.71073 Å
Hall symbol: -C 2yc	Cell parameters from 9270 reflections
*a* = 18.6899 (6) Å	θ = 2.2–29.2°
*b* = 14.4941 (4) Å	µ = 0.08 mm^−^^1^
*c* = 16.7485 (5) Å	*T* = 113 K
β = 95.266 (2)°	Block, violet
*V* = 4517.9 (2) Å^3^	0.61 × 0.53 × 0.33 mm
*Z* = 2	

### Data collection

**Table:**

Bruker-Nonius APEXII CCD diffractometer	6086 independent reflections
Radiation source: fine-focus sealed tube	4957 reflections with *I* > 2σ(*I*)
Graphite monochromator	*R*_int_ = 0.033
Detector resolution: 8.333 pixels mm^-1^	θ_max_ = 29.3°, θ_min_ = 2.4°
φ and ω scans	*h* = −25→25
Absorption correction: multi-scan (*SADABS*; Blessing, 1995)	*k* = −19→19
*T*_min_ = 0.678, *T*_max_ = 0.746	*l* = −22→23
51842 measured reflections	

### Refinement

**Table:**

Refinement on *F*^2^	Primary atom site location: structure-invariant direct methods
Least-squares matrix: full	Secondary atom site location: difference Fourier map
*R*[*F*^2^ > 2σ(*F*^2^)] = 0.047	Hydrogen site location: inferred from neighbouring sites
*wR*(*F*^2^) = 0.138	H atoms treated by a mixture of independent and constrained refinement
*S* = 1.04	*w* = 1/[σ^2^(*F*_o_^2^) + (0.0684*P*)^2^ + 3.4092*P*] where *P* = (*F*_o_^2^ + 2*F*_c_^2^)/3
6089 reflections	(Δ/σ)_max_ < 0.001
289 parameters	Δρ_max_ = 0.44 e Å^−^^3^
5 restraints	Δρ_min_ = −0.34 e Å^−^^3^

### Special details

**Table:**

Geometry. All e.s.d.'s (except the e.s.d. in the dihedral angle between two l.s. planes) are estimated using the full covariance matrix. The cell e.s.d.'s are taken into account individually in the estimation of e.s.d.'s in distances, angles and torsion angles; correlations between e.s.d.'s in cell parameters are only used when they are defined by crystal symmetry. An approximate (isotropic) treatment of cell e.s.d.'s is used for estimating e.s.d.'s involving l.s. planes.
Refinement. Refinement of *F*^2^ against ALL reflections. The weighted *R*-factor *wR* and goodness of fit *S* are based on *F*^2^, conventional *R*-factors *R* are based on *F*, with *F* set to zero for negative *F*^2^. The threshold expression of *F*^2^ > σ(*F*^2^) is used only for calculating *R*-factors(gt) *etc*. and is not relevant to the choice of reflections for refinement. *R*-factors based on *F*^2^ are statistically about twice as large as those based on *F*, and *R*-factors based on ALL data will be even larger.

### Fractional atomic coordinates and isotropic or equivalent isotropic displacement parameters (Å^2^)

**Table:**

	*x*	*y*	*z*	*U*_iso_*/*U*_eq_	Occ. (<1)
O1	0.61544 (5)	−0.22668 (6)	0.49734 (5)	0.02549 (19)	
N1	0.74054 (7)	−0.33495 (9)	0.63481 (7)	0.0354 (3)	
N2	0.81037 (8)	−0.05230 (10)	0.62647 (10)	0.0519 (4)	
N4	0.28556 (6)	0.21599 (9)	0.17128 (7)	0.0337 (3)	
N3	0.67592 (6)	0.09283 (8)	0.51591 (7)	0.0342 (3)	
C1	0.72855 (6)	−0.26329 (9)	0.60753 (7)	0.0263 (2)	
C2	0.71622 (6)	−0.17294 (9)	0.57628 (7)	0.0238 (2)	
C3	0.76699 (7)	−0.10418 (10)	0.60298 (8)	0.0318 (3)	
C4	0.56689 (6)	−0.09065 (8)	0.43879 (6)	0.0200 (2)	
C5	0.62913 (6)	−0.07176 (8)	0.48837 (6)	0.0204 (2)	
C6	0.65688 (6)	−0.15572 (8)	0.52307 (6)	0.0211 (2)	
C7	0.55438 (6)	−0.19392 (8)	0.44206 (7)	0.0216 (2)	
C8	0.56100 (7)	−0.24283 (9)	0.36256 (7)	0.0285 (3)	
H8A	0.5624	−0.3097	0.3713	0.043*	
H8B	0.6053	−0.2231	0.3405	0.043*	
H8C	0.5196	−0.2272	0.3248	0.043*	
C9	0.48701 (7)	−0.22100 (9)	0.48084 (8)	0.0286 (3)	
H9A	0.4870	−0.1905	0.5331	0.043*	
H9B	0.4862	−0.2881	0.4882	0.043*	
H9C	0.4445	−0.2019	0.4463	0.043*	
C10	0.65681 (6)	0.01838 (8)	0.50443 (7)	0.0237 (2)	
C11	0.52475 (6)	−0.02424 (8)	0.39539 (6)	0.0215 (2)	
H11	0.5409	0.0379	0.3989	0.026*	
C12	0.46183 (6)	−0.04148 (8)	0.34801 (6)	0.0206 (2)	
H12	0.4463	−0.1038	0.3441	0.025*	
C13	0.41816 (6)	0.02472 (8)	0.30455 (6)	0.0198 (2)	
C14	0.35532 (6)	−0.00359 (8)	0.25653 (7)	0.0229 (2)	
H14	0.3431	−0.0672	0.2544	0.027*	0.081 (2)
O2A	0.34030 (5)	−0.09416 (7)	0.25497 (6)	0.0295 (3)	0.919 (2)
H2A	0.3035 (10)	−0.1063 (13)	0.2199 (11)	0.035*	0.919 (2)
C15	0.31146 (6)	0.05859 (9)	0.21287 (7)	0.0273 (3)	
H15	0.2696	0.0371	0.1820	0.033*	
C16	0.32793 (7)	0.15364 (9)	0.21348 (7)	0.0268 (3)	
C17	0.39137 (7)	0.18304 (9)	0.26073 (7)	0.0270 (2)	
H17	0.4044	0.2464	0.2620	0.032*	
C18	0.43327 (6)	0.12048 (8)	0.30389 (7)	0.0230 (2)	
H18	0.4748	0.1422	0.3352	0.028*	0.919 (2)
O2B	0.4814 (5)	0.1629 (7)	0.3528 (6)	0.0295 (3)	0.081 (2)
H2B	0.5066 (16)	0.203 (3)	0.333 (3)	0.035*	0.081 (2)
C19	0.22046 (8)	0.18697 (11)	0.12259 (8)	0.0385 (3)	
H19A	0.2297	0.1275	0.0963	0.046*	
H19B	0.2085	0.2333	0.0800	0.046*	
C20	0.15686 (8)	0.17596 (13)	0.17183 (11)	0.0464 (4)	
H20A	0.1459	0.2354	0.1958	0.070*	
H20B	0.1685	0.1306	0.2144	0.070*	
H20C	0.1150	0.1548	0.1371	0.070*	
C21	0.30658 (8)	0.31313 (10)	0.16457 (9)	0.0357 (3)	
H21A	0.3280	0.3353	0.2174	0.043*	
H21B	0.2634	0.3508	0.1490	0.043*	
C22	0.36029 (10)	0.32545 (13)	0.10298 (10)	0.0483 (4)	
H22A	0.3407	0.2992	0.0517	0.072*	
H22B	0.4052	0.2938	0.1213	0.072*	
H22C	0.3698	0.3913	0.0962	0.072*	
O3	0.4975 (9)	0.3578 (3)	0.2641 (9)	0.086 (2)	0.25
C23	0.5000	0.5166 (6)	0.2500	0.105 (3)	0.50
H23A	0.5191	0.5035	0.1986	0.158*	0.25
H23B	0.5359	0.5507	0.2847	0.158*	0.25
H23C	0.4562	0.5537	0.2408	0.158*	0.25
C24	0.4839 (5)	0.4325 (6)	0.2875 (7)	0.086 (2)	0.25
C25	0.4474 (5)	0.4443 (6)	0.3616 (8)	0.086 (2)	0.25
H25A	0.4438	0.3845	0.3882	0.129*	0.25
H25B	0.3991	0.4693	0.3480	0.129*	0.25
H25C	0.4751	0.4871	0.3977	0.129*	0.25

### Atomic displacement parameters (Å^2^)

**Table:**

	*U*^11^	*U*^22^	*U*^33^	*U*^12^	*U*^13^	*U*^23^
O1	0.0254 (4)	0.0211 (4)	0.0284 (4)	0.0027 (3)	−0.0068 (3)	0.0028 (3)
N1	0.0359 (6)	0.0361 (6)	0.0331 (6)	0.0097 (5)	−0.0033 (5)	0.0076 (5)
N2	0.0411 (7)	0.0437 (8)	0.0659 (9)	−0.0064 (6)	−0.0213 (7)	0.0056 (7)
N4	0.0316 (6)	0.0376 (6)	0.0307 (5)	0.0118 (5)	−0.0044 (4)	0.0062 (5)
N3	0.0336 (6)	0.0286 (6)	0.0386 (6)	−0.0030 (5)	−0.0059 (5)	−0.0012 (5)
C1	0.0230 (5)	0.0330 (6)	0.0222 (5)	0.0067 (5)	−0.0020 (4)	0.0023 (5)
C2	0.0210 (5)	0.0278 (6)	0.0219 (5)	0.0045 (4)	−0.0020 (4)	0.0028 (4)
C3	0.0266 (6)	0.0335 (7)	0.0335 (6)	0.0049 (5)	−0.0076 (5)	0.0047 (5)
C4	0.0189 (5)	0.0216 (5)	0.0191 (5)	0.0020 (4)	−0.0009 (4)	0.0001 (4)
C5	0.0185 (5)	0.0218 (5)	0.0202 (5)	0.0024 (4)	−0.0021 (4)	0.0008 (4)
C6	0.0201 (5)	0.0231 (5)	0.0200 (5)	0.0033 (4)	0.0003 (4)	0.0008 (4)
C7	0.0200 (5)	0.0212 (5)	0.0226 (5)	0.0021 (4)	−0.0042 (4)	0.0009 (4)
C8	0.0305 (6)	0.0272 (6)	0.0270 (6)	0.0031 (5)	−0.0021 (5)	−0.0051 (5)
C9	0.0260 (6)	0.0282 (6)	0.0315 (6)	−0.0025 (5)	0.0020 (5)	0.0026 (5)
C10	0.0203 (5)	0.0267 (6)	0.0230 (5)	0.0024 (4)	−0.0033 (4)	0.0012 (4)
C11	0.0210 (5)	0.0215 (5)	0.0212 (5)	0.0022 (4)	−0.0020 (4)	0.0008 (4)
C12	0.0204 (5)	0.0218 (5)	0.0191 (5)	0.0021 (4)	−0.0010 (4)	0.0006 (4)
C13	0.0179 (5)	0.0234 (5)	0.0177 (5)	0.0030 (4)	−0.0011 (4)	−0.0005 (4)
C14	0.0206 (5)	0.0259 (6)	0.0216 (5)	0.0020 (4)	−0.0018 (4)	−0.0032 (4)
O2A	0.0274 (5)	0.0231 (5)	0.0354 (5)	−0.0005 (4)	−0.0115 (4)	−0.0025 (4)
C15	0.0207 (5)	0.0362 (7)	0.0235 (5)	0.0049 (5)	−0.0051 (4)	−0.0025 (5)
C16	0.0254 (6)	0.0329 (6)	0.0218 (5)	0.0095 (5)	−0.0001 (4)	0.0028 (4)
C17	0.0287 (6)	0.0252 (6)	0.0266 (6)	0.0036 (5)	0.0005 (5)	0.0030 (4)
C18	0.0225 (5)	0.0257 (6)	0.0200 (5)	0.0005 (4)	−0.0021 (4)	0.0003 (4)
O2B	0.0274 (5)	0.0231 (5)	0.0354 (5)	−0.0005 (4)	−0.0115 (4)	−0.0025 (4)
C19	0.0359 (7)	0.0457 (8)	0.0311 (6)	0.0147 (6)	−0.0121 (5)	0.0016 (6)
C20	0.0317 (7)	0.0537 (10)	0.0517 (9)	0.0122 (7)	−0.0083 (6)	−0.0029 (7)
C21	0.0402 (7)	0.0335 (7)	0.0332 (6)	0.0157 (6)	0.0025 (6)	0.0065 (5)
C22	0.0616 (11)	0.0467 (9)	0.0381 (8)	0.0144 (8)	0.0131 (7)	0.0114 (7)
O3	0.057 (3)	0.0470 (19)	0.148 (7)	0.013 (2)	−0.023 (4)	−0.023 (3)
C23	0.120 (7)	0.066 (5)	0.131 (8)	0.000	0.016 (6)	0.000
C24	0.057 (3)	0.0470 (19)	0.148 (7)	0.013 (2)	−0.023 (4)	−0.023 (3)
C25	0.057 (3)	0.0470 (19)	0.148 (7)	0.013 (2)	−0.023 (4)	−0.023 (3)

### Geometric parameters (Å, º)

**Table:**

O1—C6	1.3351 (14)	O2A—H2A	0.88 (2)
O1—C7	1.4802 (13)	C15—C16	1.4116 (19)
N1—C1	1.1484 (17)	C15—H15	0.9500
N2—C3	1.1485 (19)	C16—C17	1.4286 (18)
N4—C16	1.3567 (15)	C17—C18	1.3617 (16)
N4—C19	1.4630 (18)	C17—H17	0.9500
N4—C21	1.469 (2)	C18—O2B	1.313 (5)
N3—C10	1.1473 (17)	C18—H18	0.9500
C1—C2	1.4212 (17)	O2B—H2B	0.840 (10)
C2—C6	1.3803 (15)	C19—C20	1.516 (2)
C2—C3	1.4198 (18)	C19—H19A	0.9900
C4—C5	1.3929 (15)	C19—H19B	0.9900
C4—C11	1.4037 (15)	C20—H20A	0.9800
C4—C7	1.5167 (16)	C20—H20B	0.9800
C5—C10	1.4218 (16)	C20—H20C	0.9800
C5—C6	1.4256 (15)	C21—C22	1.514 (2)
C7—C9	1.5203 (17)	C21—H21A	0.9900
C7—C8	1.5235 (16)	C21—H21B	0.9900
C8—H8A	0.9800	C22—H22A	0.9800
C8—H8B	0.9800	C22—H22B	0.9800
C8—H8C	0.9800	C22—H22C	0.9800
C9—H9A	0.9800	O3—C24	1.187 (11)
C9—H9B	0.9800	C23—C24	1.416 (10)
C9—H9C	0.9800	C23—H23A	0.9800
C11—C12	1.3798 (15)	C23—H23B	0.9800
C11—H11	0.9500	C23—H23C	0.9800
C12—C13	1.4169 (15)	C24—C24^i^	1.44 (2)
C12—H12	0.9500	C24—O3^i^	1.447 (14)
C13—C18	1.4166 (16)	C24—C25	1.480 (12)
C13—C14	1.4216 (15)	C25—H25A	0.9800
C14—C15	1.3816 (16)	C25—H25B	0.9800
C14—H14	0.9500	C25—H25C	0.9800
C14—O2A	1.3422 (15)		
			
C6—O1—C7	110.24 (8)	C16—C17—H17	119.9
C16—N4—C19	120.99 (12)	O2B—C18—C17	110.3 (5)
C16—N4—C21	122.06 (12)	O2B—C18—C13	125.4 (5)
C19—N4—C21	116.58 (11)	C17—C18—C13	123.45 (11)
N1—C1—C2	177.39 (14)	C17—C18—H18	118.3
C6—C2—C3	123.41 (11)	C13—C18—H18	118.3
C6—C2—C1	119.87 (11)	C18—O2B—H2B	117 (3)
C3—C2—C1	116.72 (10)	N4—C19—C20	112.37 (12)
N2—C3—C2	176.28 (15)	N4—C19—H19A	109.1
C5—C4—C11	124.90 (11)	C20—C19—H19A	109.1
C5—C4—C7	107.19 (9)	N4—C19—H19B	109.1
C11—C4—C7	127.91 (10)	C20—C19—H19B	109.1
C4—C5—C10	124.25 (10)	H19A—C19—H19B	107.9
C4—C5—C6	109.15 (10)	C19—C20—H20A	109.5
C10—C5—C6	126.50 (10)	C19—C20—H20B	109.5
O1—C6—C2	118.49 (10)	H20A—C20—H20B	109.5
O1—C6—C5	110.33 (9)	C19—C20—H20C	109.5
C2—C6—C5	131.18 (11)	H20A—C20—H20C	109.5
O1—C7—C4	103.09 (8)	H20B—C20—H20C	109.5
O1—C7—C9	105.73 (9)	N4—C21—C22	111.34 (12)
C4—C7—C9	114.00 (10)	N4—C21—H21A	109.4
O1—C7—C8	106.11 (9)	C22—C21—H21A	109.4
C4—C7—C8	113.73 (10)	N4—C21—H21B	109.4
C9—C7—C8	112.97 (10)	C22—C21—H21B	109.4
N3—C10—C5	176.62 (13)	H21A—C21—H21B	108.0
C12—C11—C4	125.57 (11)	C21—C22—H22A	109.5
C12—C11—H11	117.2	C21—C22—H22B	109.5
C4—C11—H11	117.2	H22A—C22—H22B	109.5
C11—C12—C13	126.38 (11)	C21—C22—H22C	109.5
C11—C12—H12	116.8	H22A—C22—H22C	109.5
C13—C12—H12	116.8	H22B—C22—H22C	109.5
C18—C13—C12	124.17 (10)	C24—C23—H23A	109.5
C18—C13—C14	115.68 (10)	C24—C23—H23B	109.5
C12—C13—C14	120.15 (10)	H23A—C23—H23B	109.5
O2A—C14—C13	116.99 (10)	C24—C23—H23C	109.5
O2A—C14—C15	120.99 (11)	H23A—C23—H23C	109.5
C14—O2A—H2A	111.0 (12)	H23B—C23—H23C	109.5
C15—C14—C13	122.04 (11)	O3—C24—C23	125.3 (11)
C15—C14—H14	119.0	C23—C24—O3^i^	107.9 (8)
C13—C14—H14	119.0	O3—C24—C25	120.8 (11)
C14—C15—C16	120.95 (11)	C23—C24—C25	113.9 (7)
C14—C15—H15	119.5	O3^i^—C24—C25	137.9 (8)
C16—C15—H15	119.5	C24—C25—H25A	109.5
N4—C16—C15	121.99 (12)	C24—C25—H25B	109.5
N4—C16—C17	120.33 (12)	H25A—C25—H25B	109.5
C15—C16—C17	117.68 (11)	C24—C25—H25C	109.5
C18—C17—C16	120.20 (12)	H25A—C25—H25C	109.5
C18—C17—H17	119.9	H25B—C25—H25C	109.5
			
C11—C4—C5—C10	2.82 (18)	C4—C11—C12—C13	179.20 (11)
C7—C4—C5—C10	−176.69 (11)	C11—C12—C13—C18	−0.24 (19)
C11—C4—C5—C6	179.34 (10)	C11—C12—C13—C14	178.55 (11)
C7—C4—C5—C6	−0.17 (13)	C18—C13—C14—C15	−0.75 (17)
C7—O1—C6—C2	179.11 (10)	C12—C13—C14—C15	−179.65 (11)
C7—O1—C6—C5	−0.50 (13)	C13—C14—C15—C16	0.76 (18)
C3—C2—C6—O1	175.13 (11)	C19—N4—C16—C15	−0.26 (19)
C1—C2—C6—O1	−5.26 (17)	C21—N4—C16—C15	−173.06 (12)
C3—C2—C6—C5	−5.4 (2)	C19—N4—C16—C17	179.96 (12)
C1—C2—C6—C5	174.26 (12)	C21—N4—C16—C17	7.15 (19)
C4—C5—C6—O1	0.42 (13)	C14—C15—C16—N4	−179.80 (12)
C10—C5—C6—O1	176.85 (11)	C14—C15—C16—C17	−0.02 (18)
C4—C5—C6—C2	−179.12 (12)	N4—C16—C17—C18	179.09 (12)
C10—C5—C6—C2	−2.7 (2)	C15—C16—C17—C18	−0.70 (18)
C6—O1—C7—C4	0.37 (12)	C16—C17—C18—O2B	−169.5 (6)
C6—O1—C7—C9	−119.59 (10)	C16—C17—C18—C13	0.70 (19)
C6—O1—C7—C8	120.19 (10)	C12—C13—C18—O2B	−12.4 (7)
C5—C4—C7—O1	−0.10 (12)	C14—C13—C18—O2B	168.7 (7)
C11—C4—C7—O1	−179.60 (11)	C12—C13—C18—C17	178.87 (11)
C5—C4—C7—C9	113.99 (11)	C14—C13—C18—C17	0.02 (17)
C11—C4—C7—C9	−65.50 (15)	C16—N4—C19—C20	82.75 (17)
C5—C4—C7—C8	−114.53 (11)	C21—N4—C19—C20	−104.06 (15)
C11—C4—C7—C8	65.98 (15)	C16—N4—C21—C22	78.93 (17)
C5—C4—C11—C12	−177.89 (11)	C19—N4—C21—C22	−94.18 (15)
C7—C4—C11—C12	1.52 (19)		

Symmetry code: (i) −*x*+1, *y*, −*z*+1/2.

### Hydrogen-bond geometry (Å, º)

**Table:**

*D*—H···*A*	*D*—H	H···*A*	*D*···*A*	*D*—H···*A*
O2*A*—H2OA···N1^ii^	0.88 (2)	1.96 (2)	2.8095 (16)	162 (2)
C17—H17···O3	0.95	2.37	3.213 (13)	148
C17—H17···O3^i^	0.95	2.51	3.324 (13)	144

Symmetry codes: (i) −*x*+1, *y*, −*z*+1/2; (ii) *x*−1/2, −*y*−1/2, *z*−1/2.
